# Making health insurance responsive to citizens: learning from six low-income and middle-income countries

**DOI:** 10.1136/bmjgh-2024-018176

**Published:** 2025-05-22

**Authors:** Anas Ismail, Inke Mathauer, Patricia Akweongo, Mery Concepcion Bolivar Vargas, Sapna Desai, Dinna Prapto Raharja, Modupe Adeoti Ogundimu, Stela Stojisavljevic, Manuela De Allegri, Zubin Cyrus Shroff

**Affiliations:** 1 Alliance for Health Policy and Systems Research, World Health Organization, Geneva, Switzerland; 2 Health Financing and Economics, World Health Organization, Geneva, Switzerland; 3 School of Public Health, University of Ghana, Accra, Ghana; 4 Faculty of Economic and Administrative Sciences, Universidad de Bogotá Jorge Tadeo Lozano, Bogota, Colombia; 5 Population Council Institute, Ahmedabad, Gujarat, India; 6 Lok Swasthya SEWA Trust, Ahmedabad, Gujarat, India; 7 International Relations, Synergy Policies, Jakarta, Indonesia; 8 National Health Insurance Authority, Abuja, Nigeria; 9 Department of Social Medicine, Public Health Institue of the Republic of Srpska, Banja Luka, Bosnia and Herzegovina; 10 Department of Public Health, Faculty of Medicine, University of Banja Luka, Banja Luka, Bosnia and Herzegovina; 11 Heidelberg Institute of Global Health, Heidelberg University, Heidelberg, Germany

**Keywords:** Health systems, Health insurance, Health Services Accessibility

## Abstract

**Background:**

Many low-income and middle-income countries have introduced public health insurance systems, whereby, thanks to government subsidies, selected groups are entitled to receive insurance coverage even if not paying direct contributions into the system. These efforts towards achieving universal health coverage were often undermined by difficulties in enrolment and registration, barriers to health service utilisation or complicated rules around service packages. Governmental and non-governmental accountability initiatives have been established to overcome these barriers in order to make health insurance programmes responsive and to empower citizens. This paper examines evidence and synthesizes lessons from 20 accountability initiatives in six selected countries to understand how these achieved (or not) these goals.

**Methods:**

We systematically analysed six final reports and five published papers which were part of a multicountry research programme from 2019 to end of 2022 studying accountability initiatives. Between June 2023 and September 2024, we systematically extracted data and synthesised findings from the reports and papers based on a conceptual framework, adapted from a framework developed by Molyneux, which had been adopted by the country teams to guide their studies. We coded the extracted data and identified the content, context and process factors that enabled or hindered the accountability initiatives in achieving their intended goals. We present and discuss factors that were present in at least two initiatives.

**Results:**

Governmental initiatives were in most instances established in conjunction with the health reforms that introduced the health insurance programmes they address. Whereas some of these initiatives were effective, many were undermined by poor outreach to citizens, inadequacy of resources, conflicts of interest and power imbalances and lack of fidelity to original design. Non-governmental initiatives often emerged to fill existing gaps in government services and programmes. Many of the non-governmental initiatives had several features which helped them in contributing to citizen empowerment, and these included embeddedness in and being trusted by the local communities, flexibility in operating and reaching out to people and the underlying motivation of people working in them.

**Conclusions:**

The effective implementation of accountability initiatives requires transparency, trust-building measures, active outreach and community engagement and adequate resources. These elements can ensure that initiatives achieve their intended goal of enhancing citizens’ access to their health insurance entitlements. Further research is needed to understand how best collaboration between governmental and non-governmental initiatives can be fostered to build synergies between the two toward the achievement of common goals.

**WHAT IS ALREADY KNOWN ON THIS TOPIC**

- Recent health insurance programmes in many low-income and middle-income countries have been undermined by suboptimal outreach efforts, complicated registration and renewal processes, service utilisation barriers and low levels of digital literacy.
- Accountability initiatives have been created to enhance the responsiveness of the health insurance programmes to citizens’ needs, but evidence is scant on their effectiveness.

**WHAT THIS STUDY ADDS**

- The study examines initiatives, both those established by government mandates and those not mandated by governments, to understand their effectiveness and factors influencing their effectiveness.
- Enablers of non-governmental initiatives included their embeddedness in the community, background trust, active and flexible outreach efforts, and the motivations of the people working on the initiatives. Governmental initiatives based on digital and web-based platforms of solutions were more effective than others.
- Active citizen engagement and participation throughout the design and implementation phases, as well as outreach efforts, are vital to the effectiveness and success of accountability initiatives.

**HOW THIS STUDY MIGHT AFFECT RESEARCH, PRACTICE OR POLICY**

- Findings from this study can support governments in strengthening initiatives mandated by them and identify how to engage non-governmental organisations to complement these efforts.
- Further research can help us understand how governmental and non-governmental accountability initiatives can work together to ensure health insurance programmes are responsive to the needs of the citizens they serve.

## Introduction

 Over the past two decades, many low-income and middle-income countries (LMICs) have introduced health insurance (type) programmes as an instrument for improving financial protection in their endeavour to move towards universal health coverage (UHC). Here, we use ‘health insurance type’ as a term that encompasses health coverage programmes that are characterised by clearly identified and affiliated beneficiaries and a clearly defined benefit package with a separate purchasing agency, which may or may not be contributory-based. They use general government financing to cover the poor and other vulnerable population groups that do not contribute themselves.

Several of these new programmes delinked entitlements from making direct contributions for various population groups and, instead, received state budget transfers to cover those not contributing.^1^,^6^ Such funding arrangements were motivated by the principle of universality underpinning UHC as well as the existence of a largely informal economy and poor and vulnerable population groups in many of these countries. The establishment of these programmes has been triggered by several factors including economic growth, political transitions and an increased recognition of the role of the private sector in service delivery, though the relative weight of each of these factors varies by country setting.^7^,^9^

Despite efforts to make these health insurance type programmes more inclusive, gaps remain in terms of population coverage, service coverage and financial protection. Various reasons are at the root of these gaps. One set of reasons relates to difficulties in enrolment and registration in health insurance, particularly affecting marginalised groups. These include lack of awareness of entitlements as well as cumbersome administrative procedures to enrol, such as the need to provide extensive documentation or other proof of programme eligibility.^10^,^12^ Other reasons relate to barriers to health service utilisation (such as informal user charges or limited government health service availability) leading to higher out-of-pocket (OOP) expenditure.^13^ Finally, complicated procedures to access services, elaborate rules around what services are included and excluded from the benefit package, and limited digital literacy often prevent people (particularly the most marginalised and vulnerable) from effectively exercising their entitlements even postenrolment/registration.^11 14^

To overcome these barriers, various accountability initiatives have been put in place by governments and non-governmental/civil society organisations (NGOs/CSOs) to make insurance programmes more responsive to citizens’ needs. These initiatives include information provision, grievance redressal or assistance in understanding entitlements and navigating health services. The available literature focuses on the impacts of the health insurance programmes themselves in terms of their impacts on utilisation rates, OOP payments and catastrophic health expenditure^15^,^18^ as well as barriers to accessing the health insurance entitlements.^10 19^ However, there is a paucity of evidence examining if and how such accountability initiatives are effective in making health insurance programmes more responsive to citizens.

To address this evidence gap, a research programme was launched by the Alliance for Health Policy and Systems Research, WHO in collaboration with the WHO Department of Health Financing and Economics (HFE) in 2019 and concluded at the end of 2022. As part of the programme, research teams in seven countries (Republic of Srpska in Bosnia and Herzegovina, Colombia, Democratic Republic of Congo, Ghana, India, Indonesia and Nigeria) examined a range of accountability initiatives to understand how they have (or not) contributed to enabling citizens to access their health insurance entitlements based on a common research framework.

This synthesis paper brings together lessons learnt from 20 initiatives (both those mandated and not mandated by governments) in 6 of the 7 countries (Republic of Srpska in Bosnia and Herzegovina, Colombia, Ghana, India, Indonesia and Nigeria). We first present the theoretical framework underpinning the research undertaken by the country teams as well as the research methods. We then highlight what accountability initiatives mandated by governments—we also refer to them as governmental initiatives—intended to do and identify factors that limited these from fully achieving their intended goals. We then explore how and to what extent initiatives not mandated by governments—hereafter referred to as non-governmental initiatives—filled these gaps to make insurance programmes more responsive and facilitated access of citizens to their health insurance entitlements. We finally end with a discussion that focuses on policy implications before we draw our conclusions.

### Research framework

**Figure 1:**
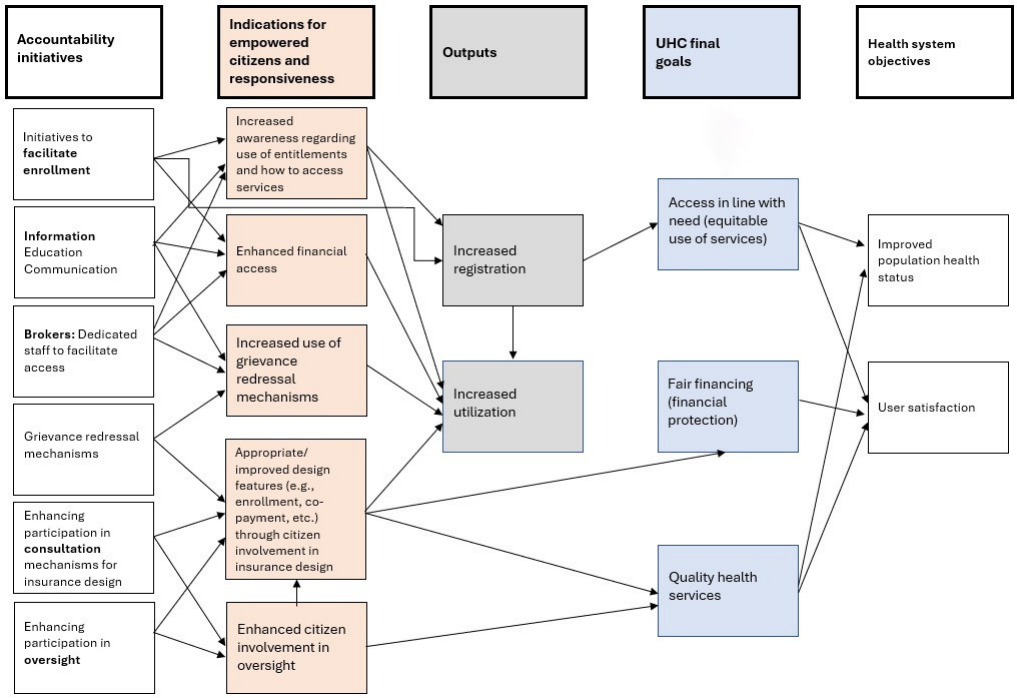
A schematic of how accountability initiatives can contribute to UHC goals and health system objectives. UHC, universal health coverage.

Figure 1 illustrates a schematic about how accountability initiatives addressing health insurance programmes might contribute to progress in UHC objectives and health system performance goals. As illustrated in the figure and shown in the initiatives we studied, these accountability initiatives serve a range of purposes. These include (a) facilitating (re)enrolment, (b) providing information on entitlements or eligibility, (c) facilitating access to health services and grievance redressal including through navigators, (d) participation in consultative processes for the design of insurance programmes and (e) enhancing insurance programme oversight. Through the multiple pathways illustrated in the figure, the effective implementation of these initiatives is expected to contribute to more responsive health insurance programmes and empowered citizens. This happens through (a) increased awareness of the health insurance programmes entitlements, (b) enhanced financial access, (c) increased use of grievance redressal functions, (d) improved design features of the programmes and (e) citizens’ oversight. As shown below, we use these indications to determine the level of effectiveness of the initiatives we studied. Increased responsivity of initiatives and empowerment of citizens would lead to higher rates of enrolment in the health insurance programme and increased health service utilisation. In turn, this is expected to contribute to the UHC objectives of increased and more equitable utilisation of health services in line with need, higher levels of financial protection and better quality of health services. These UHC objectives are indispensable elements to the achievement of the ultimate health systems objectives of improved population health and health systems responsiveness.

**Figure 2:**
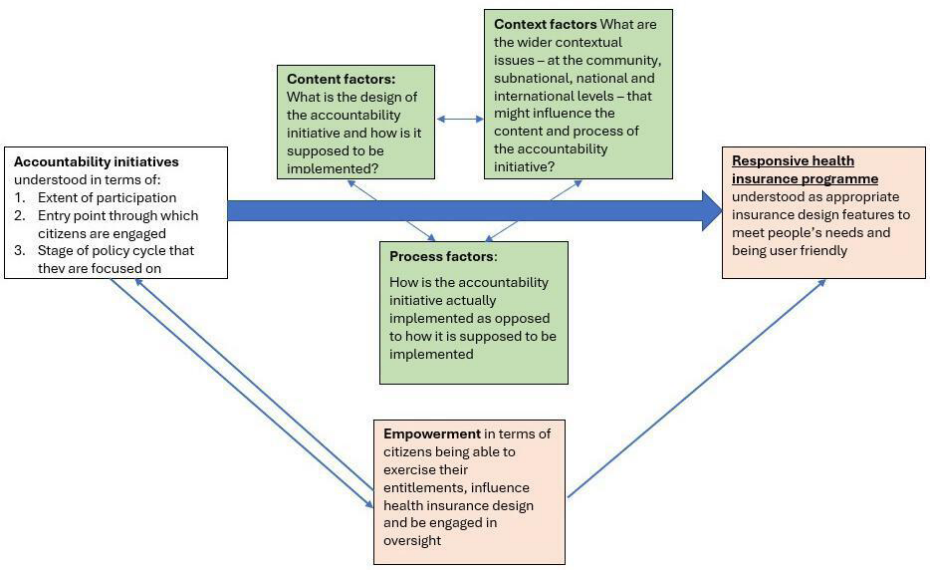
A common research framework adopted by the country team to inform their data collection and analysis.

A common research framework to inform data collection and analysis (figure 2) was developed and refined with input from country research teams through an in-person workshop at the beginning of the programme in July 2019. This facilitated a joint understanding of terms and concepts across the research teams and informed the development of data collection tools for both key informant interviews and structured surveys. This approach facilitated cross-country comparison towards drawing lessons that can be transferred to other contexts. We used a modified version of Molyneux’s framework which originally assessed community accountability at peripheral health facilities.^20^ Applied to our research programme, the framework sought to understand how the interaction of content, context and process (further explained below) influenced whether or not accountability initiatives were able to effectively contribute to increased responsiveness of publicly funded health insurance programmes as well as empowerment of citizens. We adapted the definition of responsiveness put forth by Goetz and Gaventa^21^ as ‘the extent to which a programme demonstrates receptivity to the views, complaints and suggestions of service users by changing structure, culture and service delivery to make it more appropriate for users’. As for empowerment, in the context of this research programme, we understood it in terms of citizens being enabled to: (a) exercise their entitlements, (b) influence health insurance design features and (c) be engaged in oversight as envisioned by the specific accountability initiatives.

Given the focus of this research programme, this would mean ensuring that health insurance programmes are designed and implemented in such a way that meets people’s needs. We operationalised this framework through three core questions related to content, context and process factors. With reference to our framework, content is used to indicate the initiative’s design and how it is supposed to operate. Process is used to indicate the actual functioning of the initiative and the respective relationships between various stakeholders. Finally, context includes both the macroeconomic and political factors as well as social and legal factors at different levels, which affect the functioning of the initiatives.

## Methods

For the purpose of developing this synthesis, we systematically analysed information from six country study reports submitted as part of the research programme that was launched by the Alliance for Health Policy and Systems Research, WHO in collaboration with the WHO Department of HFE in 2019 and concluded at the end of 2022. Five research teams went on to publish peer-reviewed journal articles.^22^,^26^ Study reports may be made available on reasonable request to the authors. Reports were reviewed and findings synthesised between June 2023 and September 2024. The reports and published papers included details on the health insurance programme in each country (described in detail below) and the accountability initiatives which sought to enhance both the responsiveness of these programmes and the empowerment of citizens. The reports themselves were developed following a common template that was devised by three of the lead authors of this paper (IM, MDA and ZCS) who provided technical support to the country research project including through providing ongoing feedback as the reports were developed. The reports included in-depth qualitative and quantitative findings on the evolution and functioning of the initiatives and how these were shaped by their content, context and process as outlined in the research framework mentioned above in figure 2. In addition, the synthesis was informed by insights which country research teams shared through regular meetings and periodic webinars in which they shared preliminary findings.

The four lead authors (AI, IM, MDA and ZCS) developed a set of predefined codes reflecting the conceptual framework to extract data and capture information from the country reports along the dimensions of the research framework (the coding sheet is provided in online supplemental appendix A). The lead authors extracted general information on the initiatives such as how they were established, when and by whom. The codes also helped us extract quantitative and qualitative findings reflecting reach and effectiveness of the initiatives to the extent possible, including their actual geographic and population coverage. We also extracted information on any challenges the initiatives faced and recommendations to improve them as reported by the country teams.

We captured information on the content, context and process aspects of these initiatives. Codes related to content included the initiative design, organisational structure, human resource arrangements, and population and geographic coverage. Under context, the codes related to the political, economic, social and legal context factors under which the initiatives operated. Under process, the codes related to the engagement of the initiatives with the community, civil society, government and the insurance company (if applicable) as well as the efforts to publicise the initiative. A fourth set of codes we used were related to relevant actors from government, the private sector and civil society as well as the beneficiaries of the initiatives.

After extracting the information under each set of codes from the reports, we sought to identify the content-, context- or process-related factors which enabled or hindered the initiatives from achieving their intended goals. Once data extraction was finalised, the lead authors discussed the extracted information to look for convergence and common factors as well as deviant cases. We only included factors that we found in at least two or more initiatives since the goal of this paper is to synthesise learning across the initiatives (individual country studies included in this series of articles examine factors particular to a given country in-depth). After synthesising these factors and how they influenced the initiatives’ performance, an interactive process followed whereby the lead authors shared the findings with authors from the country teams to ensure that the synthesis and its interpretation were in line with the interpretation of the country teams.

### Patient and public involvement

Patient and public involvement statements are included in three of the published articles.^22 24 26^ Given the nature of this synthesis, no patient or public involvement was done. While patients and the public did not participate in the design of the research programme, consultations were held at the beginning of the programme with technical and local experts to guide its scope and implementation. Moreover, the paper was developed with dissemination plans in mind which involve informing patients and the public, particularly those involved in the primary studies, of the findings of the synthesis.

## Results

This section describes the main findings from the cross-country analysis of the initiatives examined by the study teams in Bosnia and Herzegovina (focusing on the elderly population in the Republic of Srpska—we use Republic of Srpska hereafter), Colombia, Ghana, India (focusing on Gujarat state), Indonesia and Nigeria. We first describe the health insurance programme in each country. We then provide an overview of the initiatives mandated by governments and explore the common factors that have hindered the achievement of their stated goals/objectives. Next, we present the initiatives not mandated by governments (largely initiated and implemented by NGOs/CSOs) and analyse how they attempted to overcome gaps left by the government-mandated initiatives, the challenges they faced in their attempt to do so, and the broader limitations of the health insurance programmes that hindered the ability of citizens to make use of their entitlements.

### Health insurance type programmes

The health insurance type programmes that were part of this cross-country analysis are presented in online supplemental table 1. While all these programmes were largely funded through public financing, there was a heterogeneity in how resources were raised (eg, general tax revenue, contributions, earmarked taxes), coverage achieved, mix of public and private providers involved, and types of services (primary, secondary and tertiary care) covered.

India’s PMJAY programme was solely funded through general tax revenues. Programmes in the Republic of Srpska, Colombia, Ghana and Indonesia were funded through a mix of general government funds and contributory payments by members of the schemes (with the Ghana programme also receiving some donor support from bilateral and multilateral partners). The National Health Insurance Scheme (NHIS) in Nigeria was composed of four different programmes which were funded through contributions from four different member groups benefiting from each programme: (1) employers and employees contribute to the formal sector programme; (2) self-registered members of the voluntary programme contribute to this programme; (3) cohesive community groups of more than 1000 people contribute to the community-based programme and (4) students contribute to the tertiary institutions programme.

Other than the health insurance programme in India—all insurance programmes were open to all citizens. The entire population was enrolled in Colombia, over 96% were enrolled in Indonesia, while this was 90% in the Republic of Srpska. However, only 8.5% of the population in Nigeria was enrolled as of June 2024. In India, the programme targets approximately 40% of people, namely those households below the poverty line. In Gujarat, where the country case study was undertaken, 62% of families below the poverty line were enrolled by December 2021. In Ghana, at least one-third of the population was enrolled for all years between 2010 and 2017. Importantly, all insurance programmes covered primary, secondary and tertiary services with the exception of India’s PMJAY which only covered inpatient secondary and tertiary care with limited outpatient or day-care treatment coverage.

### Accountability initiatives mandated by governments

Table 1 provides a brief description of all accountability initiatives included in this synthesis. The initiatives mandated and implemented by governments aimed to increase citizens’ access to health services and enhance the responsiveness of the health insurance programmes. The initiatives varied in terms of their purpose. For example, the NHIS mobile platform in Ghana focused on membership renewal and, more recently, registration. Some initiatives had multiple functions including information provision, navigation support and grievance redressal services. Examples of these include the User Associations in Colombia, Protectors of Patient’s Health Insurance Entitlements (PPHIEs) in the Republic of Srpska and the NHIS desk officers (NDOs) in Nigeria.

**Table 1:** Brief description of the initiatives mandated by governments and initiatives not mandated by governments

	Country	Name and year begun	Objectives	Population covered
Initiatives mandated by governments				
1	Bosnia and Herzegovina	Protectors of Patient’s Health Insurance Entitlement (2011)	To provide one-to-one patient support, to provide information, help patients navigate the health system and receive members’ grievances.	Information provision and navigation support to the entire population in the Republic of Srpska; registered people can use grievance redressal services.
2	Colombia	Letters of Rights and Duties (LOR) and Letters of Performance (LOP) (2009)	LORs: to inform members of their rights and entitlements and of the services they can benefit from.LOPs: to inform members of how insurers performed, such as position in the official rankings of the insurers (EPSs) according to the performance review carried out by the MOH, accreditation, financial indicators. The LOPs endeavoured to elicit competition among the insurers.	These initiatives covered almost all the population of Colombia as 96% are covered by the health insurance programme.
3		User Associations of the Insurance Companies (1993)	To enable members to represent themselves before the EPSs and protect their rights through oversight of quality and implementation of services.	
4		Citizen Involvement in priority setting exercises (1993)	To enable members to participate in deciding the services that are included in or excluded from the health insurance benefit package.	
5	Ghana	The NHIS Mobile Renewal Service Platform (2019)	To ease the membership renewal process for enrolees without having to visit an NHIS office.	All NHIS members were covered, except groups fully exempted from paying premiums who must renew their membership through district offices.
6		The Grievance Redressal Mechanism (2004)	To allow members to lodge their complaints about exercising their health insurance entitlements	The initiative targeted all people registered with the NHIS.
7	Indonesia	Lapor.go.id (2009)	To allow members to lodge complaints and provide feedback and suggestions around government programmes in general. JKN is one of the programmes that is included in the scope of this website.	The initiative targeted all those using public services within Indonesia.
8		BPJS Kesehatan Care Centre (2014)	To provide members with a one-stop-shop for information provision (membership, payments, empanelled hospitals) and receiving complaints.	The initiative targeted all people enrolled in the JKN health insurance.
9		Mobile JKN (2017)	To integrate various services (eg, registration, changing membership data, making payments, filling grievances) in one mobile application to increase efficiency.	The initiative targeted all JKN members; there were 11.3 million downloads by the end of 2020.
10		BPJS Satu (2020)	To provide information and support to patients directly at the health facility and receive their complaints.	The initiative targeted all people enrolled in the JKN health insurance.
11	Nigeria	NHIS Desk officers (NDOs) (2005)	To provide direct assistance to patients in hospitals through giving them information, helping them navigate health services and facilitating onsite grievance redressal.	NDOs were staff of NHIS liaising with employees of government organisations.
12		Central Government Ministries, Departments, and Agencies Desk Officers (MDOs) (2008)	To provide assistance to members who are government employees through arranging meetings with health insurance officers.	MDOs were staff of government organisations who liaised between NHIS and their organisations.
13		Customer care and call centre (2016)	To provide support to members through providing them with information about their entitlements and receiving and elevating their grievances.	All members of the NHIS.
14		Subnational offices (2013)	To provide registration and complaint mechanisms for citizens, convening stakeholders together and elevating major infractions of service providers to the head office.	All members of the NHIS.
Initiatives not mandated by governments				
1	Bosnia and Herzegovina	Family Medicine Teams (2001)	To help the elderly utilise their health insurance entitlements by giving them relevant information and helping them navigate the health system.	The entire population of the Republic of Srpska is covered by the initiative as they are all registered with family medicine teams.
2	India	SEWA Shakti Kendras (SSKs) (2015)	To provide SEWA members with one-stop centres that offer multiple services within the co Smmunity.To facilitate SEWA members‘ access to and utilisation of health insurance entitlements.	The initiative covers SEWA women members working in the informal economy. In June 2024, there were 39 SSKs covering around 30 000 households.
3	Indonesia	BPJS Watch (2014)	To provide direct support to citizens in using their health insurance entitlements.	The initiative operates in five regions in Indonesia and targets the poor.
4		Jamkeswatch (2014)	To provide direct support to citizens in using their health insurance entitlements.	The initiative has offices in all provinces in the country and has approximately 2000 activists. The initiative targets the poor and patients with low education levels.
5		Posko JKN-KIS (2015)	To provide direct support to citizens in using their health insurance entitlements.	The initiative helps patients in the Bekasi region.
6	Nigeria	CSOs and Board of Trustees for the Mutual Health Associations (MHAs) (2012)	To allow beneficiaries of the MHAs to have a say in the design and monitoring of the programme.To provide information to beneficiaries and facilitate their access to care.	The initiative targets members of the MHAs and informal and community-based insurance programmes.

BPJS, Badan Penyelenggara Jaminan Sosial Kesehatan (Social Security Agency on Health); CSOs, civil society organisations; JKN, Jaminan Kesehatan Nasional (National Health Insurance); KIS, Kartu Indonesia Sehat (Indonesia Health Card); NHIS, National Health Insurance Scheme; SEWA, Self-Employed Women’s Association.

The range of initiatives mandated by governments indicates a strong policy interest to enhance citizen access to services, enhance accountability of these programmes to those they were established to serve and potentially give citizens more say in how these programmes were designed and implemented. This was also reflected in the high number of governmental initiatives established to improve the functioning of the health insurance programmes in their pursuit of UHC. However, in several settings studied, the implementation of the initiatives has not always lived up to these promises. We examined some of the factors that explain this gap between policy interest and uneven, often sub-optimal implementation.

Table 2 provides indications of reach and effectiveness of the governmental initiatives and highlights some of the challenges they faced. The table presents the main quantitative and qualitative findings from the primary country studies to which we allude below while discussing the content, context and process factors that hindered or enabled the initiatives from achieving their intended goals. As for the extent of reach of the initiatives, it varied between, and even within, countries. Some initiatives, such as the mobile renewal service in Ghana and the BPJS Kesehatan Care Centre in Indonesia, reached higher percentages of their intended populations than others. One of the main challenges was the poor outreach efforts leading to a lack of citizen awareness of the initiatives and challenges in availing their support services. For example, in the Republic of Srpska, the PPHIEs, who are mandated to both provide information and grievance redressal, were known to only 4% of the surveyed elderly. Similarly, in Colombia, 24.6% and 2.8% of 1311 surveyed households knew about the Letters of Rights and Duties (letters sent by the insurers to inform their members of their rights and duties) and Letters of Performance (letters sent by the insurers to their members to inform them how the insurer is performing relative to other insurers), and 8.8% and 0.7% used them, respectively. Citizens in Colombia also did not seem to understand the intent or process to realise their right to be involved in priority-setting exercises for the health insurance scheme.

**Table 2:** Reach, effectiveness and challenges of the initiatives mandated by governments

	Country and name	Indications for reach	Indications for effectiveness	Challenges
1	Bosnia and Herzegovina, PPHIE	The elderly (target group of the study) generally lacked awareness of the initiative and its functions (4% were aware of it).	Only 2 out of 11 PPHIE employees reported spending some time at the health centre to perform the intended tasks of the initiative.	Little awareness among the elderly of the PPHIE and their functions. Shortages in resources prevent PPHIEs from deploying to clinics and so unable to carry out their intended function.
2	Colombia, LOR and LOP	24.6% and 2.8% of households surveyed by the study (n=1311) remembered receiving the LOR and LOP, respectively.	8.8% and 0.7% of households surveyed by the study used the LOR and LOP, respectively.	Laws governing the content of the letters are contradictory and hard to follow. Complying with all laws makes the letters complicated and very long (median of 100 pages and 40 000 words).
3	Colombia, User Associations of the Insurance Companies	70% of the 1117 municipalities in Colombia had user associations; 56% of health insurance members had an operational user association in their municipality	10% of the surveyed households knew of any user association, 3% had communicated with one and 1% found them useful.	Lack of resources to form and run user associations. Little efforts are made to promote the user associations. Generational/age gap between people running the user associations (retirees and older people) and people benefitting from them (mostly younger generations)
4	Colombia, Citizen Involvement in priority setting exercises	8.7% of surveyed households signed up for the MOH priority setting website ‘Mi VOX Populi’ and 2.2% took part in a priority settings process.	Regulatory loopholes and powerful influence of the private sector and pharmaceutical industry reduced the effectiveness of citizens’ involvement	Limited and disadvantaged citizens involvement compared with private sector. MOH doesn’t empower active citizen participation. Not all services of the health insurance programme were included in the priority setting exercises.
5	Ghana, The NHIS Mobile Renewal Service Platform	In all 12 FGDs and 90/108 of the in-depth interviews, participants were aware of the renewal platform; 60% of surveyed respondents had a mobile wallet account and could use the platform.	Around 69% (n=1099) of surveyed households with active membership renewed their membership using the mobile service; 21% of the population is illiterate and depend on mobile money vendors to use the platform; vulnerable groups (children, pregnant women, indigent) could not use the platform and must present in person for renewal	The renewed membership status sometimes was not verified nor reflected on the system in the health facility. Mobile network problems delay the renewal process.
6	Ghana, The Grievance Redressal Mechanism	There was little awareness about the toll-free number as people used the district office to lodge their complaints.	34 persons (5.6%) reported grievances through the initiative out of 609 who reported experiencing grievances while accessing healthcare; 16 of the 34 received feedback.	Little awareness of the grievance redressal mechanism and of the toll-free number. Grievance redressal team claims that members don't provide all requested information limiting their ability to deal with the complaints.
7	Indonesia, Lapor.go.id	None of the JKN patients and navigators interviewed in the study (n=54) were aware of the initiative’s relevance to JKN nor have they used it before.	Whereas the website received 1000–1200 complaints daily, 40% of these are shelved or unprocessed; a total of 712 complaints throughout 2019 were for health insurance.	Insufficient number of staff (15) manage the initiative, and a third (5/15) are unpaid and without professional training for the job. JKN is not a priority for the website initiative as it is government wide and covers all ministries and districts. Not all bodies relevant to JKN have access to the JKN-relevant data from the website.
8	Indonesia, BPJS Kesehatan Care Centre	All JKN members interviewed (n=18) in the study were aware of it.The centre had two offices with 111 staff, and they receive 5000–7000 calls during the weekdays and 1000–2000 during the weekends.	JKN members who used the services said, ‘it is just a formality’ and had low trust in it.	Lack of knowledge among the JKN members about the benefits of JKN, which complicated the work of the centre. There was a lag in updating the membership database with other relevant databases or when members paid their contribution through other agencies which limited the ability of the care centre to effectively serve as a ‘one stop shop’.
9	Indonesia, Mobile JKN	As of May 2023, around 24 million users downloaded the application.	It was the third most common source of information after BPJS Kesehatan Care Centre and social networks (word of mouth).JKN members report that information is sometimes incorrect or hard to find on the application, which limits its use.	JKN members could not use the mobile application if there are mistakes or discrepancy in their membership data. The information available on the application was not always updated with real-time information, such as bed availability in hospitals, which was not helpful when solving real-time barriers.
10	Indonesia, BPJS Satu	One officer was assigned to one big hospital (receiving more than 15 000 patients each month) and 5 other smaller hospitals.	Officers did not have the time to attend to all cases in the 6 hospitals to which they’re assigned.The initiative is more used by subsidised and non-salaried JKN members, who ask about membership status, payments, referrals and hospital bed availability.	Satu officers had limited access to members’ data which delayed providing services to the members and caused unnecessary out-of-pocket payments. A considerable time was wasted on transportation between the hospitals, making officers prioritise the large hospital they cover over the smaller ones.
11	Nigeria, NHIS Desk Officers (NDOs)	A fourth of the study sample (n=654) met with the NDOs in the health facilities.	Nearly 80% of people in the study who contacted the NDOs found them useful.	Extremely insufficient HR capacity and frequent absenteeism. No involvement of beneficiaries in design of the initiative. Lack of supervision of NDOs and of response from Head office to issues raised by NDOs.
12	Nigeria, MDOs	Over half the participants (n=654) had contact with MDOs before.	75% of participants who contacted MDOs found them useful.	High turnover of MDOs resulting from frequents transfers. The high turnover resulted in loss of institutional memory and constant need for training and retraining.
13	Nigeria, Customer care and call centre	Nearly half (42.6%) of participants (n=654) knew they could channel grievances through the call centre.	About 20% of participants had actually used the call centre for grievance redressal. Nearly two-thirds of these users found the centre helpful in resolving their complaints.	Due to lack of automation at the centre, there was no recording of the calls received, making follow-up difficult.
14	Nigeria, subnational offices	75.1% of study participants (n=654) were aware they can use the offices for grievance redressal.	51.5% of participants actually visited the offices to lodge complaints for grievance redressal, and nearly 90% of these found the offices helpful in solving their grievances.	The offices lack authority to sanction healthcare providers and are underfunded. Delays in communication between the Head Office and the subnational offices.

BPJS, Badan Penyelenggara Jaminan Sosial; FGDs, Focus Group Discussions; HR, Human Resources; JKN, Jaminan Kesehatan Nasional; LOP, Letters of Performance; LOR, Letters of Rights; MDOs, Ministries, Departments and Agencies Desk Officers; NHIS, National Health Insurance Scheme; PPHIE, Patient’s Health Insurance Entitlements.

Another major challenge was the inadequacy of resources to implement the initiatives as designed. For instance, although the Lapor.go.id website in Indonesia aimed to provide grievance redressal for a range of government programmes/functions, there were only 15 staff allocated to work on this initiative, a major limitation for a country as vast as Indonesia. Moreover, one-third of the staff were unpaid volunteers. A similar situation was noted with the NDOs in Nigeria, whose numbers only covered a small fraction of the health facility in a country with a large population such as Nigeria. This made the NDOs feel they have to prioritise some health facilities and leave out others, as noted by one of them, ‘so which hospital do we go to, and which ones do we leave out?’. Another set of challenges pertained specifically to initiatives that relied heavily on digital technology. These included low levels of digital literacy among the intended users, such as in the Mobile JKN in Indonesia, as well as delays in transmitting information, such as renewal status of beneficiaries in the mobile renewal service in Ghana. Following this overview, we present below the findings and common aspects in relation to the content, process and context of the initiatives that enabled or limited them in achieving their intended objectives.

#### Content

The content and design of the government-mandated initiatives were not always conducive to achieving their intended targets. Two main factors that emerged from the analysis were: (mis)alignment of the initiative design with its intended functions, and conflicts of interests that were often built into the initiatives.

In some cases, the design of government-mandated initiatives did not facilitate their ability to achieve their envisioned function. For example, initiatives that were tasked with processing grievances (eg, PPHIEs in the Republic of Srpska, and NDOs and national and subnational offices in Nigeria) had no authority to contribute to or influence the grievance redressal process. The role of staff working in these initiatives was thus reduced to receiving grievances and transmitting them, impeding the ability of the initiative to meaningfully address them. In other cases, the design did not meaningfully consider the needs of target groups. In Colombia, various laws mandated the design (ie, content) of the Letters of Rights and Duties and Letters of Performance without standard guidelines around making them clearly readable and succinct. As a result, the Letters did not achieve their intended functions as they were difficult to comprehend given their length (a median length of 100 pages) and complex language that included legal terms. Similarly, in Ghana, membership renewal through the mobile phone application excluded, by design, children, pregnant women and the indigent who were still mandated to renew their insurance membership manually, resulting in a seemingly needless barrier to renewal for these vulnerable groups.

Moreover, the design of several initiatives was riddled with conflicts of interest that potentially made implementation of accountability mechanisms largely tokenistic. The Health Promotion Entities (EPSs, in Spanish), the health service purchasing agencies in Colombia, were mandated by law to organise user associations that would supposedly scrutinise the work of the EPSs. The EPSs were also mandated to send out the Letters of Rights and Duties and the Letters of Performance to inform the citizens of their rights and of the quality of the services of the EPSs. This design created strong incentives for the EPSs to merely tick boxes of what is mandated by law rather than implement the initiatives effectively. Thus, the letters were difficult to read and the user associations were poorly resourced so that only 3% of the people surveyed in the primary study communicated with a user association and 1% found them useful. Other examples included grievance redressal initiatives in Indonesia and the Republic of Srpska where the staff who processed grievances were themselves employees of the health insurance programmes, making it difficult for them to hold relevant programmes to account.

#### Process

The processes, that is, the way the initiatives were realised in practice, played a crucial role in influencing their effectiveness and ability to respond to citizen needs. Three key process factors that emerged from the analysis included: lack of implementation fidelity to original design, poor availability of human and financial resources, something that often underlay the lack of implementation fidelity, and lag in real-time provision of data from databases and networks hampering digital initiatives.

The initiatives were often not implemented as designed for various reasons. The PPHIE in the Republic of Srpska were expected to spend at least 50% of their time in hospitals to enable patients to directly access them at facilities. Given the shortage of resources, PPHIE staff were burdened with administrative roles that limited their presence in hospitals and thus the ability of patients to access them. A similar trend was seen with *Satu* officers in Indonesia who each were assigned to cover a big hospital (receiving over 15 000 patients) plus five smaller hospitals, making it extremely hard for the officers to keep fidelity to the original design of the initiative they were implementing due to time constraints. Thus, their presence at more peripheral health facilities was compromised with negative consequences for patient access to their services. Citizen’ involvement in priority-setting exercises in Colombia is another example. Although the initiative’s design involved including citizens’ input in the biennial exercise to include or exclude services and devices covered by the insurance programme, the actual implementation of these exercises did not allow for much citizen involvement. Patients were consulted close to the end of the priority setting exercises, just before decisions were adopted and published. This made citizen input marginal when compared with other influences, for example, inputs from pharmaceutical companies and the private sector. Furthermore, some health technologies were included in the insurance package through mechanisms that completely bypassed the citizen input stage, further undermining the initiative.

The availability of adequate human and financial resources is a crucial prerequisite for the effective implementation of initiatives, and this was clearly inadequate in several initiatives. The user associations in Colombia were self-funded and were not provided resources to meet or train their leaders and representatives on how they can effectively contribute. This, coupled with the fact that these were set up by EPSs (which often had little interest in them being effective), resulted in these associations being poorly staffed with little to no financial resources. As a result, in many instances, their functioning was reduced to a tokenistic engagement. The Health Insurance Fund (HIF) in the Republic of Srpska lacked adequate resources to sufficiently staff the PPHIEs to carry out the full range of tasks they were mandated to do. This resulted in them shifting staff (including those working within facilities) to perform HIF administrative roles. Also, the HIF provided few resources to train PPHIEs for their roles in the hospital while engaging with patients. Similar issues were faced by the NDOs in Nigeria, who experienced challenges on account of inadequate staffing and an absence of supervision.

A third factor was related to the four mobile phone-based or internet-based initiatives found in Indonesia and Ghana. The functioning of these initiatives was hindered by delays in real-time availability of data and problems in synchronisation of databases on which the initiatives were based. In Ghana, people who renewed their membership through the mobile platform often did not have their active membership status reflected in the health facilities in a timely fashion. As such, they could not access health services when they needed them. Similarly in Indonesia, the utilisation of the Mobile Jaminan Kesehatan Nasional (JKN) initiative was impeded in cases of discrepancies in personal data imported to the mobile platform from national registries or by gaps in some features of the application that prevented users from using them. Moreover, the database on the mobile platform was not updated with real-time information on available hospital beds, such that patients could not rely on this function of the mobile phone app when they sought an available bed. Also in Indonesia, launching a grievance via the website lapor.go.id sometimes did not lead to any concrete action on account of not all relevant government bodies having access to JKN data. This meant that grievances launched through the website in many instances never reached the concerned body or authority.

#### Context

We identified three contextual factors as important in influencing the reach and effectiveness of governmental initiatives. These factors were related to literacy and familiarity with digital technology among target populations, beneficiary expectations and apprehensions around questioning authority, and power imbalances between actors responsible for implementing the initiatives at different levels.

Poverty and illiteracy (including a lack of digital literacy) hindered the uptake of initiatives which relied substantially on digital technology for their everyday implementation. For instance, mobile-based initiatives were introduced in Ghana and Indonesia to enable people to not have to make the physical journey to health insurance programme offices to renew their membership and pay premiums, get information about the programme and lodge complaints about problems that they faced. However, considerable numbers of people in both countries had neither the digital literacy nor the digital connectivity required to use these. In Ghana, this spurred the growth and involvement of middlemen referred to as ‘mobile money merchants’ who charged people a fee to use mobile-based initiatives through their devices. Similarly, the Letters of Rights and Duties and the Letters of Performance in Colombia were mostly available in digital formats on the internet. Thus, in addition to these letters being challenging to read (as highlighted in the content section above), access to them was also limited on account of users not knowing where these letters were to be found.

Beneficiary attitudes, whether shaped by historical legacies or current realities, created a context that in many instances was counter-productive to the effective implementation of initiatives. Bosnia and Herzegovina’s long history of conflict resulted in older populations in the Republic of Srpska having low expectations from the health system and being easily satisfied with the health services they were provided. The fear of authority also made them anxious to not be seen as critical or complaining, which they believed might lead them to being singled out as troublemakers, in turn affecting their receipt of health services. Moreover, in Ghana, Indonesia and Colombia, the studies revealed that people had little trust in the grievance redressal initiatives put in place and believed that using these initiatives would not help address the challenges they faced. In Ghana, for example, while 42.6% of the 654 people surveyed knew they could channel their grievances through the customer care and call centre, only 20% of those surveyed actually did so. Instead, people resorted to complaining verbally or using the personal phone numbers of National Health Insurance Agency (NHIA) staff to address their problems, an approach which resulted in their being more immediately listened to than going through what were perceived as more bureaucratic channels.

Power imbalances between involved actors at different levels of implementation were noted to impede the work of some initiatives. This was most clearly seen in Nigeria, where subnational offices had less power than the head office whose staff had the power to take over and implement the work plans originally drawn by the subnational office staff. This created friction between the two levels as well as between the NDOs who actually implemented the initiative and the head offices, which resulted in delays in communications between the different levels. Another example was seen in Colombia, where the EPSs overall did not provide user associations with resources to work (such as a room, communication means and some finances). This resulted in power imbalances as the user associations could not effectively carry out the accountability functions they were supposed to do, with many associations serving as mere symbols of community participation and empowerment.

### Accountability initiatives not mandated by governments

Information on the six initiatives not mandated by governments in four countries analysed in this cross-country synthesis is provided in table 1. These include *SEW*A’s (Self-Employed Women’s Association) Resource Centre (SEWA Shakti Kendras, SSK) in Gujarat, India, informal navigation support groups in Indonesia (the BPJS Watch, Jamkeswatch and Posko JKN-KIS initiatives), the Family Medicine Teams (FMTs) in Republic of Srpska and the board of trustees (BOTs) of the Mutual Health Associations (MHAs) in Nigeria. The majority of these initiatives emerged organically through community-based organisations or through individuals that engaged with community members and patients in the delivery of health services.

SSKs in Gujarat are community-based resource centres which were launched by the Health Team of SEWA, which is a trade union of informal sector women workers, to assist their members with accessing health entitlements and broader government services. The three initiatives in Indonesia are similar in that they emerged from union activists who sought to help people in their communities access their health insurance entitlements. As for the FMTs in the Republic of Srpska, while located within public facilities and staffed by public sector employees, they were included here as patients also used them as information providers and navigators in practice to fill the gaps left by the PPHIEs who were mandated to perform these functions. The BoTs of the MHAs initiative in Nigeria were introduced to increase people’s participation in the community-based health insurance scheme through participation in its design, information provision to its beneficiaries, and monitoring its implementation. In Ghana, mobile merchants emerged as a market response to people’s inability to enrol and renew digitally on their own but were not considered an organised initiative in any sense and thus were not included here. We refer to it where relevant in some of the findings below.

Table 3 shows the reach and effectiveness of the initiatives not mandated by government. Compared with the governmental initiatives, people in general appeared to be more aware of these initiatives. This could be attributed to their embeddedness in their communities and/or long-term community activism. In this section, we present the common aspects of these initiatives that enabled them to move closer to achieving their intended objectives but also factors that limited them. We analyse these aspects along the content, context and process, as was done for the government mandated initiatives.

**Table 3:** Reach and effectiveness of the initiatives not mandated by governments

	Country and name	Indications for reach	Indications for effectiveness
1	Bosnia and Herzegovina (Republic of Srpska), Family Medicine Teams (FMTs)	99% of surveyed elderly were registered with FMTs.	96% of surveyed elderly had confidence in the FMT they’re registered with.The FMTs were the most common source of information on health insurance for the elderly.
2	India, SEWA Shakti Kendras (SSKs)	SEWA had 39 SSKs in Gujarat by June 2024. The 6 centres studied had 12 730 contacts with beneficiaries over 14 months, which included awareness raising through home visits, group meetings, and visits to health facilities; nearly half of the contacts were related to health insurance.	The women reported increased awareness around health insurance through gaining valuable information from contacts with SEWA workers, such as health insurance entitlements and registration and documentation as well as health issues overall.Home visits ensured inclusiveness of disabled and marginalised women.The exposure visits to health facilities were particularly effective in empowering women to access services independently.
3	Indonesia, BPJS Watch	Activists were available to the beneficiaries through phone and WhatsApp; activists advocated for the rights of the beneficiaries regularly in the media.	The initiative activists helped 4–6 cases each month.
4	Indonesia, Jamkeswatch	Around 1000 activists were available to the beneficiaries through phone and WhatsApp; activists advocated for the rights of the beneficiaries regularly in the newspapers and social media.	The activists felt they were known and trusted at the hospital they served; a few patients reported being confident repeating the process the activists facilitated for them.
5	Indonesia, Posko JKN-KIS	The initiative was run through WhatsApp which started as a group of acquaintances who had JKN issues and later grew so patients who needed help could get in touch; the WhatsApp group had 217 volunteers who received, on average, 5 cases per day.	Only a small fraction of the interviewed patients who received help from informal navigators were confident that they could navigate JKN independently after receiving help from the initiative.
6	Nigeria, CSOs and Board of Trustees (BoT) for the Mutual Health Associations (MHAs)	The initiative was designed with little input from the beneficiaries, whereas the BoTs felt the beneficiaries asked too much compared with the premiums they paid.	Overall, the initiative has some positive contributions, but its contribution to the beneficiaries’ empowerment and responsiveness was relatively low due to many challenges facing the work of the BoTs of the MHAs, including shortage of resources, cultural and religious practices, and beneficiaries channelling complaints informally through community leaders rather than BoTs.

CSOs, civil society organisations; SEWA, Self-Employed Women’s Association.

#### Content

These initiatives often emerged or were designed to help overcome access barriers in the health insurance programmes or fill gaps in government mandated initiatives. When examining across these initiatives, two content factors emerged as having contributed to their effectiveness: the broad range of support services provided and the embeddedness of the people delivering the services within the communities they served.

The support services offered to the beneficiaries beyond health insurance-related assistance were associated with a higher level of awareness of these initiatives among communities. In Gujarat, India, for example, women saw the SSK centres as a ‘one-stop shop’ where they could seek assistance with multiple concerns, from understanding PMJAY health insurance entitlements to hands-on support in accessing health facilities and obtaining information on health and nutrition. The elderly in the Republic of Srpska viewed the FMTs in a similar way, trusting them to be both providers of healthcare and medical advice and a reliable source of information on their health insurance entitlements. The range of services provided in both cases (beyond only information or grievance redressal) ensured more people benefited from these initiatives which, in turn, generated greater trust and demand from the community, leading to increased penetration and ultimately effectiveness of the initiatives.

The people and volunteers who implemented these initiatives were also more likely to be embedded in the communities and had experience serving them over a long period of time. This helped them reach their target groups to offer support. The various navigation support initiatives in Indonesia emerged from long-standing community or labour activists, who were well-known to the communities around them. The involvement of these well-known activists, as well as their appearance on the media and their writing in the newspapers, helped motivate hundreds of activists to be available on WhatsApp groups to respond to patients’ requests and offer their help. This made it easy for people to contact them and ask for help and guidance when having difficulties accessing their health insurance entitlements. Similarly, the SSKs in India and FMTs in the Republic of Srpska also had long-standing relations with the local populations which they had maintained over the years. Women working in SSK centres were themselves SEWA members, and the centres were designed to be within the communities’ reach. Additionally, SSK staff engaged in home visits to their beneficiaries to ensure that hard-to-reach people also received the services they needed. The FMTs in the Republic of Srpska were close to and had strong links to the communities who relied on them for primary healthcare services, particularly the elderly who almost universally were registered with an FMT as reported in the country case study.

#### Process

How the non-governmental initiatives were implemented was closely linked to their design. By nature, these initiatives were less formal and in many instances less structured than governmental initiatives. Factors that enabled effective operation included the use of communication technology and innovation, including overall flexibility in the way they operate, and the underlying motivation of those working in these initiatives.

The NGOs/CSOs implementing the initiatives showed great flexibility in the way they worked, enabling communities to engage with their services. WhatsApp was the main means of communication for the initiatives in Indonesia, both between the patients and the volunteers and between the volunteers themselves. Moreover, the volunteers in the initiatives in Indonesia would also provide navigation support on the ground to patients and not just support them through WhatsApp. Similarly, the FMTs in the Republic of Srpska kept themselves abreast of changes in health insurance entitlements and offered this information directly to patients while they sought healthcare from the FMTs.

To be able to provide services to their beneficiaries, the non-governmental initiatives often relied on establishing and maintaining good relations with the authorities and health service providers. In India, for instance, SEWA had a long history of engaging with local governments on various issues. Existing relations with the authorities helped them effectively partner with service providers to support government programmes, including around enabling people to access health insurance entitlements. On the other hand, the volunteers of the initiatives in Indonesia could count on a long history of activism and work in unions. While their attitude towards government ranged from very cooperative to somewhat confrontational, what was overarching was the desire to ensure that people got access to their entitlements. Volunteers working in these initiatives continuously followed the changes in the JKN to be able to assist their beneficiaries in the best way possible. In the case of the BoTs of the MHAs in Nigeria, the reach and effectiveness were undermined by the absence of political will and support which prevented the proper implementation of the initiative.

#### Context

The contexts in which the non-governmental initiatives operated contributed to them being able to reach local communities and move closer to their goals. First, one of their main rationales (when they emerged) was to fill gaps in existing government services, either in the health insurance programmes or in terms of the functioning of existing government accountability initiatives. Second, the initiatives enjoyed a pre-existing trust resulting from the longstanding work of the organisations that developed them within the community which facilitated their work.

Local organisations identified gaps in government services and initiatives that shaped efforts to support communities in accessing and using entitlements. The inadequacy of PPHIEs in providing information and supporting system navigation functions led to FMTs being contacted by patients for these services. Another example is the emerging market for mobile merchants in Ghana to help people with the mobile renewal applications who lack the digital literacy to use the application or who have no mobile wallets to pay through the application. Given that only 60% of the population had mobile wallets and that around 20% are illiterate, the mobile phone application established by NHIA for enrolment left a considerable gap. This allowed the mobile merchants to emerge in response to those people wishing to make use of the convenience of the mobile renewal platform, but unable to use the digital interface on their own.

The non-governmental initiatives operated in a context of a strong pre-existing trust in the organisations that developed them that was built over the years. In the Republic of Srpska, for instance, 96% of the elderly surveyed in the case study saw the FMTs as trustworthy. This finding was very much as a result of years of providing services to the elderly and the community in general. In India, SEWA’s long-standing work organising women workers facilitated effective expansion into improving access to and use of health insurance, with a focus on approaches that were responsive to women’s needs. Similarly, the non-governmental initiatives in Indonesia were born out of a long history of labour activism, and the leaders of the initiatives were trusted figures in the community, which further validated the initiatives and their services. Moreover, the SSK staff as well as the navigators in Indonesia actively invested in maintaining partnerships with government. This facilitated their work and allowed them to deliver services to and assist their beneficiaries, further boosting their trustworthiness in the communities around them.

## Discussion

This synthesis generated evidence from studying 20 accountability initiatives in six countries. It revealed that many of the government-mandated initiatives examined in this synthesis (see table 1) were undermined by inadequate levels of awareness among beneficiaries, lack of resources and suboptimal design and implementation, including conflicts of interests and improper allocation of resources. The synthesis also revealed how the non-governmental initiatives sought to address these gaps by building on the long-standing relation and trust with communities, showing flexibility and agility in implementation, responding to community needs through offering a range of support services, and building and maintaining relations with government bodies and authorities.

Examples from the literature on participatory governance show that active and meaningful participation of community members in decision-making, including scrutinising the policies and decisions of healthcare providers and insurers, leads to improved health services and health outcomes.^27 28^ Such meaningful participation was largely missing in the majority of the governmental initiatives we studied. For example, user associations and the Letters of Rights and Duties and the Letters of Performance in Colombia were largely ineffective as they were more concerned with satisfying the legislative requirements than really engaging citizens in insurance design and oversight. Rather than carefully thinking about how initiative design might best ease access to entitlements and enhance accountability, governmental initiatives often seemed to be about ticking boxes and being somewhat tokenistic, with little done to incentivise effective action and redress power imbalances. To overcome these weaknesses, it is critical to redesign accountability initiatives, particularly governmental ones, to overcome conflicts of interests and power imbalances across different system levels in a way that allows for greater citizen participation and oversight.

One factor that limited citizens from benefiting from their full health insurance entitlements was the limited outreach or focus on ensuring citizens are aware of the scope of the insurance programmes, coverage, services offered and how to access them. The government-mandated initiatives, which were often built into the health insurance programmes and in conjunction with the reforms, aspired to empower citizens to benefit from the full range of health services covered by the health insurance programmes. However, they were not consistently able to build awareness because outreach efforts were not integrated into the initiatives or because of inadequate resources which did not allow the staff to properly advertise the initiatives. This is not unique to the countries we studied. A study of health facility governing committees which were mandated by the government in Tanzania showed ineffective outreach efforts which resulted in considerably low levels of awareness and participation in the committees.^29^ Limited citizens’ awareness of accountability initiatives and their health insurance entitlements can lead to worse outcomes in relation to UHC and health systems objectives (see figure 1 above). Evidence from India showed increased OOP expenditure by patients covered by PMJAY as private hospitals sometimes took advantage of the citizens not knowing what they are entitled to and charging them for services for which the hospitals will be reimbursed by PMJAY.^30^ On the other hand, people were more aware of the non-governmental initiatives, which were embedded in communities and accordingly used a range of approaches to ensure awareness such as home visits, in-person support at health facilities, and social media communication tools. Community outreach and raising awareness through diverse communication channels are two modalities through which governmental initiatives can potentially improve their effectiveness and reach among different groups in the community.

Pre-existing trust and embeddedness in the community were significant drivers of the ability of non-governmental initiatives to be effective, particularly in terms of addressing grievances. A recent review of studies on community engagement in primary healthcare underscored the importance of building robust relations and trust with the community as important factors to the success of community engagement efforts.^31^ Two of the studies in the review looked at Aboriginal Australians: one on their engagement in designing and delivering healthcare through District Aboriginal Health Action Groups^32^ and another on their participation in consultations to develop primary healthcare services.^33^ One of the elements that led to the effectiveness of the implementation of the first initiative was the continuous building of trust through the community engagement process. This in turn meant that aboriginal communities were actively involved in decision-making processes and, as a result, increased their trust and utilisation of the health services being provided to them. In the second initiative, genuine and extensive community engagement resulted in high levels of trust in the local healthcare providers, which in turn played an important role in ensuring the effective implementation of the initiative. Also, evidence shows that when it comes to people’s choices of their insurers and healthcare providers, such choices are largely influenced by how much trust they have in the insurer purchasing the services they need and the provider offering them high quality services.^34 35^ In the initiatives we studied, we noted that people did not use grievance redressal mechanisms when they did not trust these mechanisms and thought they were futile or would cause them trouble. Evidence from a recent review of the literature on grievance redressal in LMICs points to similar observations where grievance redressal mechanisms were not able to achieve redress, which resulted in low levels of trust in, and subsequently utilisation of, these mechanisms.^36^ On the other hand, people relied on support from SSKs in addressing challenges related to utilisation or on grassroots initiatives in Indonesia, whose leaders were long-term activists, several of whom regularly appear in the media.

The shortages in financial and/or human resources made it difficult for some governmental initiatives to keep fidelity to their original design. This was seen with PPHIE officers in the Republic of Srpska, with Satu officers in Indonesia, with NDOs and subnational offices in Nigeria, and with staff working for lapor.go.id in Indonesia. In these cases, employees were either inadequate in number, overstretched across facilities much beyond their capacity, lacking in training or forced to task shift to cover other roles. Resource shortages are a very common factor widely cited in the literature as a challenge to implement initiatives to empower communities as originally envisaged.^28 31 37^ For example, similar to the resource constraints facing user associations in Colombia, health committees which were set up as a means of community participation in primary healthcare in South Africa faced limited financial resources and lack of training which hindered their role and effectiveness.^37^ Moreover, given that many health insurance programmes and accountability initiatives in our synthesis were created in conjunction with reforms, the shortages in resources could reflect poor planning of resources required to fully and effectively implement the reforms. Another example from South Africa illustrates this point, where plans to implement national health insurance and primary healthcare re-engineering did not effectively address shortages and maldistribution of human resources across provinces, eventually undermining the re-engineering efforts.^38^ Alternatively, in the context of the initiatives in our study, it may potentially reflect an overall low priority accorded to these initiatives.

Digital tools have become widely used in the health sector,^39 40^ and also increasingly to support health financing reforms.^41 42^ Digital tools were employed both by governmental and non-governmental initiatives with some success, but there also were several problems that hindered them achieving their full potential. The digital technologies in the initiatives supported various functions and tasks, from registration and re-enrolment in the programmes to information provision and communicating patients’ grievances. Some of the problems limiting their effectiveness related to limited digital literacy and mobile phone and internet access of certain population groups, with the likelihood of increasing the digital divide across the populations. Another set of problems related to inadequate synchronisation of databases, access to data and limited interoperability between different databases required to undertake these health financing tasks for delivering health insurance entitlements. Such issues are common to many uses of digital technologies in the health sector in general, as discussed by Abernethy *et al*^40^ and in the domain of health financing,^41^ particularly with health insurance programme management.^43^ Countries should, therefore, consider designing and deploying digital technologies in a way that takes account of digital divides between various population groups to address inequities and avoid their further increase. Abernethy *et al*^40^ present what they believe are requirements for digital health infrastructure, and among them are equitable access and engagement of individuals with the technology, keeping with data standards and curation protocols, interoperability of different mediums of digital technologies and recordkeeping, and support enhancing the skills of the workforce to skilfully use digital technologies. Furthermore, digital technologies should be seen as one set of tools to enhance the functioning of health insurance programmes and solve some of their problems. Digital technologies by themselves cannot address the systemic gaps and inadequate design features of health insurance programmes, particularly in LMICs.

Non-governmental initiatives demonstrated agility and attention to the context and needs of their target groups, something that often set them apart from governmental initiatives that could be administratively more cumbersome in some cases. For example, grievance redressal through government officials involved filling out forms and waiting for long processes via peripheral and central administrative offices, and vulnerable groups in Ghana could not use the mobile renewal platform and were required to present themselves physically to renew their memberships. On the other hand, SSKs in India and volunteers in Indonesia could offer immediate help through various means to people who contacted them in lodging complaints or accessing health services. There are several aspects through which governmental initiatives can improve their agility and flexibility: easing their procedures, conferring more power to employees in peripheral offices to provide practical assistance to people, incentivising those who work in governmental initiatives to provide needed assistance for vulnerable groups, and collaborating with grassroots and non-governmental groups which could facilitate communication between people and government services. Furthermore, the discrepancies between the two types of initiatives provide an area for potential synergy where governmental initiatives can make use of implementation support from non-governmental initiatives, which by nature are more agile and flexible. One example of such synergy is India’s Village Health Sanitation and Nutrition Committees, a governmental initiative to enhance community governance in health, whose implementation was revitalised with the help of NGOs.^44^

The findings we presented in this synthesis provide evidence that can be useful to health financing policy-makers and programme implementers. To start, policy-makers can support a culture of accountability within health insurance programmes by incorporating the views of citizens in the design and implementation of such initiatives. The Colombian case study in our synthesis was the only case in the countries and initiatives that we studied where efforts were made to provide for citizen participation in insurance design and oversight, designed to set up participation options for citizens. Yet, this comes along with the need to provide adequate resources to such initiatives in order to allow citizens to be represented and to actively and effectively participate in the design of health policies. Furthermore, not only should participation be an integral part of designing and implementing policies and programmes, but initiatives should also seek improvements based on feedback from the users. Experiences from South Korea^45^ and Thailand,^46^ for example, demonstrate the critical role of engaging citizens and civic groups when formulating health system, particularly health financing, reforms.

Overall, this paper, and the case studies on which it builds, highlights key features of accountability initiatives within publicly-funded health insurance type programmes in several settings. One consistent finding across the initiatives was the central role of citizen engagement and participation to ensure that potential users are aware of their entitlements and how to use them and have the capacity to access health services. The paper showed gaps in outreach to and engagement with citizens which significantly undermined citizens’ ability to use such initiatives. Information provision and awareness raising are thus important policy measures needed. Furthermore, the synthesis underscores the importance of accountability initiatives, including trust-building measures, that are needed to contribute to the effective implementation of health insurance type programmes that seek to move countries closer to UHC. Last but not least, it is important to endow these accountability initiatives with adequate resources for them to operate effectively.

As far as limitations are concerned, this synthesis relied on data collected by the country research teams for their primary studies and, thus, it did not collect new data to directly inform the comparison. This was mitigated by using publicly available data to update information on coverage and enrolment figures for the health insurance programmes and the initiatives to ensure the synthesis is as up to date as possible. Second, the initiatives involved in the synthesis were purposively identified and selected by the country research teams for their studies, and so they do not necessarily represent the full range of existing accountability initiatives within each country. Such an approach also does not enable us to compare one government-mandated and non-government-mandated initiative in each setting, which would have been ideal. Third, while the teams applied a common research framework, the studies did not have a common protocol in terms of exact questions asked. This was deliberate to allow country teams flexibility in terms of developing questions suited to their context and to the specific aspect of the accountability initiative that they were examining in detail for their country-based research article. This limitation was mitigated by involving country authors in this synthesis piece, particularly in interpreting findings extracted from the country studies.

## Conclusions

In conclusion, accountability initiatives can play an important role in contributing to making health insurance programmes more responsive to citizens and empowering citizens they intend to serve. This synthesis was based on experiences from six countries, where accountability initiatives had variable reach and effectiveness, with some being hardly known to participants or largely ineffective and others being very well-known or quite effective in helping citizens access their health insurance entitlements. The synthesis offered detailed insights into content, context and process factors that impacted the effectiveness of accountability initiatives in these countries. We learnt that even when accountability initiatives are designed with good intentions, their implementation needs to be thought through thoroughly to ensure they have adequate resources to be properly implemented, that they incorporate citizens’ views and feedback, they seek trust-building measures, and they actively reach out to communities to make beneficiaries aware of them. Increased collaboration between government-mandated initiatives and non-government ones can be useful to identify complementarities and strengthen both types of initiatives towards improved health. A future area for research is to explore examples of such collaboration and understand best practices that make these collaborations more effective in driving health insurance programmes to be more responsive to the citizens they serve.

Nonetheless, as much as the accountability initiatives are important, we are cognisant that the accountability initiatives in and of themselves cannot fix issues in health financing policies or weak design and implementation of health insurance programmes. In other words, they are not enough to rectify inadequate health financing policy design or address access problems even when these initiatives are effective. Although citizen participation and accountability and responsiveness arrangements have to be pursued at the centre of health coverage scheme design, ultimately, in order to make progress towards UHC, health financing policy needs to reflect desirable health financing attributes, be informed by evidence about what has worked in health financing and be realised in adequate design and implementation of health insurance programmes.

## Supplementary material

10.1136/bmjgh-2024-018176 online supplemental file 1

10.1136/bmjgh-2024-018176 online supplemental file 2

10.1136/bmjgh-2024-018176 Uncited online supplemental material 1

## Data Availability

Data are available on reasonable request.
