# Supplementary material for: Making health insurance responsive to citizens: learning from six low-income and middle-income countries
Source: BMJ Glob Health. 2025 May 22;7(Suppl 6):e018176. doi: 10.1136/bmjgh-2024-018176 (PMC12107628; doi:10.1136/bmjgh-2024-018176)
Supplement: online supplemental file 2 [file bmjgh-7-Suppl_6-s002.docx]

Supplementary Table 1. Brief description of the health insurance programmes.

| Country | Name and year of inception | Target group & actual coverage | Benefits | Revenue source | Health service providers |
| --- | --- | --- | --- | --- | --- |
| Bosnia and Herzegovina (Republic of Srpska) | Health Insurance Fund (1996) | The programme targets all people in the Republic of Srpska; 90% of the population is covered. | Primary, secondary, tertiary services, transportation and healthcare at home. | Contributions from the insured and government funds. | Mainly government institutions. Limited number of health services provided at private health care institutions. |
| Colombia | The Contributory and Subsidized Health Insurance Schemes (1993) | 96% of people in Colombia enrolled through either the Contributory scheme or the Subsidized scheme. | Both schemes cover primary, secondary, and tertiary services. | 12.5% contribution from salaried employees. Of this 12.5%, 11% funds the Contributory scheme and 1.5% helped fund the Subsidized Scheme, which receives additional support from a solidarity fund paid through national and local tax. | Public and private sector; insurance companies are not required to contract public sector in the Contributory Scheme but are required to do so for the Subsidized Scheme. |
|  |  |  |  |  |  |
| Ghana | National Health Insurance Scheme (2004) | All people targeted. The NHIS covered 95% of disease burden in the country; enrolment in the scheme was 33% ,41% and 35% in 2010, 2015, and 2017 respectively. | Primary, secondary, and tertiary services. | The Central National Health Insurance Fund from contributions, yearly premiums, levy on taxes, and donor support. Several groups of people are exempt from contribution. | Public and private sector. |
| India | Pradhan Mantri Jan Arogya Yojana (2018) | People living below the poverty line are the target population (the bottom 40% of the population); coverage was 62% of eligible families in Gujarat (the focus state for the study included in this programme) by end of 2021. | Hospitalization and selected outpatient services. | General government revenues. | Public and private sector. |
| Indonesia | National Health Insurance Programme (Jaminan Kesehatan Nasional) (2014) | All citizens in Indonesia targeted; coverage was 96.06% in April 2024. | Primary, secondary and tertiary services. | National and local government subsidies for subsidized members (nearly 60% of members); contributions from employer and employee (totaling 5% of salary); direct payments for self-employed for each individual in their families. | Public and private sector; services covered in private sector are reviewed annually. |
| Nigeria | National Health Insurance Scheme^[[1]](#footnote-1)^ (2004) | All people in Nigeria are targeted through at least one of four programmes. These programmes are for a) formal sector employees, b) voluntary contributors, c) students in tertiary educational institutions and d) community based programmes for households or individuals willingly pooling risks together. Overall coverage was about 8.5% (n= 18,675,088) in June 2024. | Primary, secondary and tertiary services. | Members of each programme make contributions to that programme. Employers contribute to the formal sector programme. Private sector employers can enroll their employees in the voluntary contributors’ programme. Community-based programmes are funded by groups of individuals, households, or occupation-based groups. | Public and private sectors. |

1. The National Health Insurance Scheme in Nigeria has become in 2022 the National Health Insurance Authority [↑](#footnote-ref-1)
