# Supplementary material for: Making health insurance responsive to citizens: learning from six low-income and middle-income countries
Source: BMJ Glob Health. 2025 May 22;7(Suppl 6):e018176. doi: 10.1136/bmjgh-2024-018176 (PMC12107628; doi:10.1136/bmjgh-2024-018176)
Supplement: Uncited online supplemental material 1 [file bmjgh-7-Suppl_6-s003.docx]

**Reflexivity Statement**: This synthesis is derived from data based on studies conducted in 6 LMICs, with each study responding to their respective local context, needs and priorities. Teams of in-country researchers (beyond the lead researchers who are also co-authors of this synthesis) led the conceptualization, design, development, implementation, and analysis of the country studies on which this synthesis builds. The conceptual framework that is referred to in the paper was co-created with the country research teams. While four lead authors (AI, IM, MDA, ZCS) led the synthesis, lead country authors contributed to this synthesis by providing country-specific information and validating emerging results and their interpretation, including the recommendations offered in the manuscript. The authors represent diversity in terms of seniority, gender, national origin and countries of residence. An early career researcher (AI) led the extraction of data from the country research reports and contributed significantly to the analysis of the data and the writing of the manuscript. In terms of gender, two of the authors (AI and ZCS) are males and eight (IM, PA, MBV, SD, DPR, MAO, SS, MDA) are females. Four authors (AI, IM, MDA, ZCS) are based in HICs. Two of the HIC based authors (AI and ZCS) are LMIC nationals with lived-experience of serving in LMIC health systems. Six authors (PA, MBV, SD, DPR, MAO, SS) are based in LMICs.
